# Data Resource Profile: The COloRECTal cancer data repository (CORECT-R)

**DOI:** 10.1093/ije/dyab122

**Published:** 2021-07-12

**Authors:** Amy Downing, Peter Hall, Rebecca Birch, Elizabeth Lemmon, Paul Affleck, Hannah Rossington, Emily Boldison, Paul Ewart, Eva J A Morris

**Affiliations:** 1 Cancer Epidemiology Group, Leeds Institute of Medical Research at St James’s and Leeds Institute for Data Analytics, University of Leeds, Leeds, UK; 2 Edinburgh Cancer Research Centre, University of Edinburgh, Western General Hospital, Edinburgh, UK; 3 Edinburgh Health Economics, University of Edinburgh, NINE BioQuarter, Edinburgh, UK; 4 Nuffield Department of Population Health, Big Data Institute, University of Oxford, Oxford, UK

**Key Features**

- CORECT-R provides an extremely secure, but researcher accessible, environment in which datasets relevant to colorectal cancer in the UK are efficiently linked, curated and analysed. It aims to overcome the significant challenges and duplication associated with access to administrative datasets and drive the production of intelligence to improve outcomes from the disease.
- Ethical approval to establish CORECT-R as a research database was obtained in 2018. Currently, the resource predominantly contains secondary administrative datasets relevant to the English colorectal cancer population. Expansion to cover the whole of the UK is under way.
- CORECT-R aims to capture detailed diagnostic, management and outcome information about all UK individuals diagnosed with, or at risk of developing, colorectal cancer. It currently contains wide-ranging information on more than 600 000 tumours. The confidentiality and security of the data within the resource is paramount and extensive procedures are in place to protect the information.
- CORECT-R has been developed by the UK Colorectal Cancer Intelligence Hub. Funded by Cancer Research UK, it is a collaboration between academics, clinical experts, patients and the public. Visit [https://www.ndph.ox.ac.uk/corectr] or contact [crchub@leeds.ac.uk] to obtain more information on how to access CORECT-R, collaborate with the Hub team and investigate questions of interest.

## Data resource basics

### Why was the resource established?

Colorectal cancer is a major public health problem in the UK. Each year in the UK around 41 000 people are diagnosed with the disease, 16 000 die from it^1^ and international comparisons indicate that survival rates are lower than those attained by our economic neighbours.^2^^,^^3^ It is estimated that detecting and managing the illness costs the National Health Service (NHS) in excess of £1.1 billion annually.^4^^,^^5^ Despite this outlay, there remain major variations in diagnosis, treatment and outcomes.^6–8^ In parallel, the research community invests significant resource and effort into understanding the aetiology of the disease and developing more effective and efficient methods of detecting and managing it.

**Figure 1:**
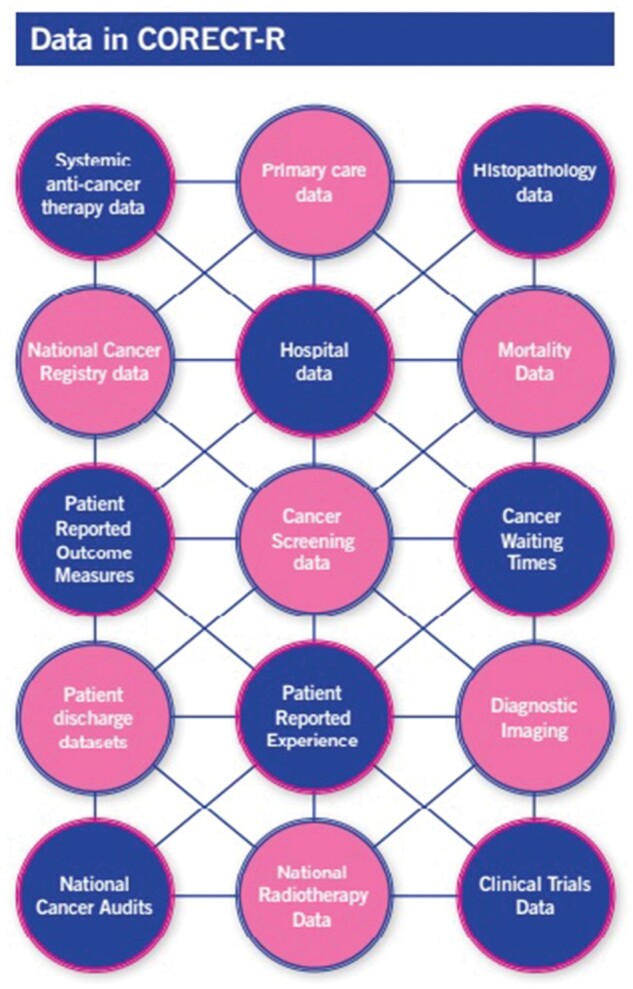
Types of data available within CORECT-R.

High-quality data are essential to improving outcomes. Good intelligence underpins patient choice, helping individuals reduce the risk of disease and access the best care. It identifies and quantifies inequalities, improves the cost-effectiveness and quality of services and supports cancer research. Unfortunately, although there are numerous individual datasets containing important information about the disease and its management, both across the UK nations and internationally, the availability of high-quality cancer intelligence has been limited due to the challenges researchers face in gaining access to them.^9^ The UK Colorectal Cancer Intelligence Hub has sought to address these data access challenges by creating a single UK colorectal cancer research data system as a model for data-driven research into all cancer types. Working in partnership with the public, patients, carers, data providers and data users, we have created a single research repository, known as the COloRECTal cancer data Repository (CORECT-R). This resource is seeking to house all the colorectal cancer data described in Figure 1 and make them more readily accessible to researchers, while ensuring the security of the data to protect patient confidentiality. In this way, the UK Colorectal Cancer Intelligence Hub seeks to generate the intelligence needed to promote early diagnosis, optimize treatment, support clinical research and, ultimately, improve the UK’s colorectal cancer outcomes.

At present, the resource contains information on all individuals diagnosed with colorectal and anal cancer in England between 1997 and 2018. This includes information on more than 600 000 cases of the disease. Much more remains to be done, however, and by incorporating more colorectal datasets from across the UK, and potentially beyond, the UK Colorectal Cancer Intelligence Hub aims to provide an annually updated and richer resource dedicated to research. This will enable far more pertinent analyses than have previously been possible.

The CORECT-R resource, and analyses based upon the data within it, has received approval from the South West-Central Bristol research ethics committee (18/SW/0134).

## Data collected

In the UK, large population datasets already exist which contain detailed information on all cancer patients. For example, in England the National Cancer Registration and Analysis Service (NCRAS) curates high-quality national cancer registration datasets that contain information on every tumour diagnosed in the country.^10^ Via linkage, or potential linkage, of these data to other administrative health datasets, such as inpatient and outpatient activity (Hospital Episode Statistics—HES),^11^ radiotherapy data (the National Radiotherapy Dataset—RTDS), cancer medicines prescribing (the Systemic Anti-Cancer Therapy—SACT),^12^ mortality data (Office for National Statistics) and many other similar datasets, there already exists a rich linked cancer data resource. Public health organizations have also built similar high-quality resources in Wales,^13^ Scotland^14^ and Northern Ireland.^15^ What has not happened routinely, however, is curation of these datasets at a disease level by academic and disease experts to realize the full potential of the information within them. Furthermore, it has often been extremely difficult for such experts to access the datasets, not least as the data are so detailed that access must be restricted to protect the confidentiality of the people the data pertain to.^16^ The CORECT-R model seeks to address these challenges by enabling dedicated data managers to work with the linked datasets within the secure digital environments, known as ‘Safe Havens’, of the respective jurisdictions.^14^^,^^15^^,^^17^^,^^18^ In this setting, the data managers act as colorectal cancer data experts and focus on the processing and management of the relevant datasets to enhance their utility and availability. This can be by, for example, deriving new variables from linked datasets and resolving quality assurance problems such as conflicting versions of the same data item arising from different data sources. Having dedicated data managers working within approved secure environments limits access to the most sensitive data items, by enabling the production of de-identified ‘research ready’ datasets for use by the cancer intelligence community.

The types of data hosted and/or linked in CORECT-R can be categorized into four main groups: routine cancer datasets, routine non-cancer datasets, consented datasets and biological samples. These groups will be described briefly here, but further details of the datasets available can be found in the CORECT-R data catalogue [https://www.ndph.ox.ac.uk/corectr/corect-r].

The first group, routine cancer datasets (such as cancer registry, patient discharge and treatment datasets), are already held within CORECT-R. These datasets provide high-quality information on many aspects of the patient pathway, including initial cancer diagnosis, treatments received and subsequent outcomes, and are providing strong intelligence to inform and improve NHS colorectal cancer services.^7^^,^^19–21^

The second group CORECT-R hosts is routine administrative datasets that contain information on people without a diagnosis of cancer. Examples of such ‘non-cancer’ data would be information on individuals participating in a screening programme, who are not identified as having cancer or prescription information from a population of people without cancer but who are comparable in terms of age and sex to those who do.^22^ These data are important, as many of the studies using CORECT-R will be strengthened by information on a comparable population without cancer. For example, studies looking to generate evidence to strengthen diagnostic pathways will require information on all who undergo the relevant diagnostic tests and not simply those who go on to get cancer. Similarly, to study the full impact of colorectal cancer on a population it is important to make comparisons with those without colorectal cancer. Linkage to non-cancer datasets is, therefore, essential to gain the intelligence needed to significantly improve colorectal cancer outcomes. The non-cancer datasets will contain no direct patient identifiers (being incorporated and linked via a secure pseudonymized process^23^), meaning that members of the CORECT-R team and users of the resource will be unable to ascertain the identity of those individuals without cancer.

The third group is consented datasets. A huge number of colorectal cancer research studies are undertaken in which individuals have consented to participate, including randomized clinical trials, cohort studies and surveys. Such studies often capture very detailed data (including study outcome measures, patient-reported outcomes and genetic information) that go beyond what is available in routine datasets, and so their inclusion has the potential to strongly enhance elements of the data. A specific example of such data already incorporated into CORECT-R is the patient-reported outcomes data taken from a national survey of colorectal cancer patients.^24^^,^^25^ CORECT-R does not always hold the additional data or samples from these consented studies, but linkage facilitates access to the relevant components of the CORECT-R data and these associated studies. Where feasible and the necessary approvals allow, individuals within CORECT-R who have consented to such studies are flagged. Over time, linkage to more consented datasets will be facilitated, but this is dependent on the approvals under which study data were collected.

Finally, CORECT-R also enables linkage to samples. Access to biological samples increases the scope of colorectal cancer research by enabling phenotype to be related to management and outcomes. CORECT-R does not host these samples directly but flags cases where these data are available. This enables identification of relevant cases and access for research projects (again, where all relevant ethical and information governance approvals are in place). An example of such data is the pathology specimens being collected through the Yorkshire Cancer Research Bowel Cancer Improvement Programme, where digital images of tumour sections are linked to the routine cancer datasets held in CORECT-R.

Researchers can seek to access all the datasets within CORECT-R in their standard or linked format. In addition however, key pieces of information will be extracted from these component datasets to form national colorectal and anal cancer datasets. These are intended to be ‘research ready’, pseudonymized population-based datasets that researchers can easily access. They avoid the need for users to independently request broader extracts of data and replicate linkages to obtain commonly used key information (for example stage at diagnosis, comorbidity scores or treatment information). These datasets are continually developing and full details are available on the Hub website [https://www.ndph.ox.ac.uk/corectr]. Tables 1 and 2 summarize their current content.

**Table 1:** Variables in the National Colorectal Cancer Dataset within CORECT-R. Currently, these relate to the English population but work is underway to expand to cover the UK

Data item	Description of field content	Format	Source of information
Pseudonymized person ID	Unique study specific identifier for each patient	Number	Derived by the UK Colorectal Cancer Intelligence Hub
Pseudonymised tumour ID	Unique study specific identifier for each tumour	Number	Derived by the UK Colorectal Cancer Intelligence Hub
Age	Option to derive age information in a format suitable for the research project needs. Can be single age or summarized in age bands (e.g. 5-year) and can also include month and year of birth	Number	Cancer registration dataset
Sex	0 = not known, 1 = male, 2 = female, 9 = not specified	Number	Cancer registration dataset
Ethnic group	Option to group ethnicities (e.g White/non-White/unknown). Several methods of deriving ethnicity information have been used previously and can be derived as per user requirements	Text	Derived by the UK Colorectal Cancer Intelligence Hub from information in the cancer registration and hospital discharge datasets
Socioeconomic status	Option to include an appropriate indicator of socioeconomic status such as the Index of Multiple Deprivation	Number	Derived by the UK Colorectal Cancer Intelligence Hub from information in multiple datasets
Vital status of the patient	A, alive; D, dead; X, exit posting	Char(1)	Cancer registration dataset
Days from diagnosis to death	Time in days between specified colorectal cancer diagnosis and death or censoring	Number	Derived by the UK Colorectal Cancer Intelligence Hub from information in the cancer registration dataset
Days from another event to date of death	Option to provide number of days from another event to death (e.g. days from diagnosis to death). Derived as per user requirements	Number	Derived by the UK Colorectal Cancer Intelligence Hub from information in key events from different datasets
Cause of death	Option to derive summary cause of death information based on the causes of death listed on death certificates or coded by former regional registries. Derived as per user requirements	Text	Cancer registration dataset
Place of death	Option to derive place of death based on information recorded on death certificates or from Hospital Episode Statistics Derived as per user requirements		Cancer registration dataset
Diagnosis date	Option to derive diagnosis date in a format suitable for the research project needs. This is likely to be in the format MM/YYYY unless permissions for access to identifiable data have been granted		Cancer registration dataset
Days from another event to date to diagnosis	Option to provide number of days from another event to diagnosis (e.g. days from birth to diagnosis). Derived as per user requirements	Number	Derived by the UK Colorectal Cancer Intelligence Hub from information in multiple datasets
Basis of diagnosis of the tumour	0 = death certificate, 1 = clinical diagnosis made before death without (2-7), 2 = clinical investigation, 4 = specific tumour markers, 5 = cytology, 6 = histology of metastases, 7 = histology of a primary tumour, 9 = unknown	Number	Cancer registration dataset
Route to diagnosis	1 = GP referral, 2 = 2-week wait pathway, 3= emergency presentation, 4 = other outpatient, 5 = screen detected, 6 = inpatient elective, 7 = death certificate only, 9 = unknown	Number	Cancer registration dataset
Site of neoplasm (4-character code)	Valid 4-digit ICD-10 codes in the range C180 to C20	Char(4)	Cancer registration dataset
Site of neoplasm (3-character code)	Valid 3-digit ICD-10 codes in the range C18 to C20	Char(4)	Cancer registration dataset
Site of neoplasm (coded)	1 = right colon, 2 = left colon, 3 = colon, unspecified, 4= rectosigmoid, 5= rectum	Number	Derived by the UK Colorectal Cancer Intelligence Hub from information in the cancer registration dataset
Morphology	The original 5-digit ICD-03 morphology information captured by NCRAS	Char(5)	Cancer registration dataset
Morphology group	A grouped morphology variable derived by grouping the detailed morphology information available in NCRAS to provide the more useable histology types of adenocarcinoma etc. For many analyses it may be appropriate to limit cases to exclude rare morphological subtypes with non-standard treatment or unusual behaviour	Number	Derived by the UK Colorectal Cancer Intelligence Hub from information in the cancer registration dataset
Summary stage	A summary stage, that combines information from the multiple staging variables available in NCRAS to maximize the number of cases with a stage classification	Number	Cancer registration dataset
Staging information	Option to derive staging information from any of the data items available in the CORECT-R tables (likely to be predominantly NCRAS Tumour table). This can include individual components of TNM or any other information that can be reported alone or combined as demanded by the research project. Derived as per user requirements		Derived by the UK Colorectal Cancer Intelligence Hub from information in the cancer registration dataset
Comorbidity scores	Option to access a number of different comorbidity scores (such as Charlson, Elixhauser, C3) created as per user requirements. This could include using a previously published application of these scores or bespoke algorithms as demanded by the research project	Number	Derived by the UK Colorectal Cancer Intelligence Hub from information in multiple datasets
Primary procedure	Primary procedure for this individual, 1 = major resection, 2 = minor resection, 3 = bypass, 4 = stoma, 5 = stent, 6 = no surgery, 7= no link to HES so no information available	Number	Derived by the UK Colorectal Cancer Intelligence Hub from information in the cancer registry and relevant hospital discharge dataset
Primary procedure OPCS4 code	The OPCS4 code indicating the exact procedure determined as the primary event	Char(4)	Hospital discharge dataset
Date of primary procedure	MM/YYYY	Number	Hospital discharge dataset
Method of admission to hospital for primary procedure	Derived from the HES variable admission method, 0 = elective, 1 = emergency, 99 = unknown	Number	Hospital discharge dataset
Location patient admitted from	1 = Unsupervised accommodation, no care provided, 2 = hospital, 3 = supervised accommodation, health care is provided, 4 = supervised accommodation, no care provided, 99 = unknown	Number	Hospital discharge dataset
Urgency of surgery	0 = elective 1 = emergency	Number	Derived by the UK Colorectal Cancer Intelligence Hub from information in the relevant hospital discharge dataset
Destination patient discharged to after primary procedure	1 = unsupervised accommodation, no care provided, 2 = hospital, 3 = supervised accommodation, health care is provided, 4 = supervised accommodation, no care provided, 9 = patient died, 99 = unknown	Number	Hospital discharge dataset
Days between diagnosis and primary procedure	Option to provide number of days from another event to diagnosis (e.g. days from birth to diagnosis). Derived as per user requirements	Number	Derived by the UK Colorectal Cancer Intelligence Hub from information in the cancer registry and relevant hospital discharge dataset
Interval between primary procedure and death or when censored	Option to provide number of days from another event to date of death (e.g. days from birth to diagnosis). Derived as per user requirements	Number	Derived by the UK Colorectal Cancer Intelligence Hub from information in the cancer registry and relevant hospital discharge dataset
Length of stay in days in the spell of care associated with the primary procedure	Total length of stay in hospital associated with the primary procedure	Number	Derived by the UK Colorectal Cancer Intelligence Hub from information in the relevant hospital discharge dataset
Length of postoperative stay in hospital	Length of post-primary procedure stay in hospital in days	Number	Derived by the UK Colorectal Cancer Intelligence Hub from information in the relevant hospital discharge dataset
Emergency readmission within 30 days of discharge following primary procedure	0 = no, 1 = yes	Number	Derived by the UK Colorectal Cancer Intelligence Hub from information in the relevant hospital discharge dataset
Death within 30 days of primary procedure	0 = alive 1 = dead	Number	Derived by the UK Colorectal Cancer Intelligence Hub from information in the cancer registry and relevant hospital discharge dataset
Death within 90 days of primary procedure	0 = alive, 1 = dead	Number	Derived by the UK Colorectal Cancer Intelligence Hub from information in the cancer registry and relevant hospital discharge dataset
Death during hospital stay of primary procedure	0 = no 1 = yes	Number	Derived by the UK Colorectal Cancer Intelligence Hub from information in the relevant hospital discharge dataset
NHS Trust for primary procedure		Char(5)	Derived by the UK Colorectal Cancer Intelligence Hub from information in the relevant hospital discharge dataset
Multidisciplinary team for primary procedure		Char(5)	Derived by the UK Colorectal Cancer Intelligence Hub from information in the cancer registry and relevant hospital discharge dataset
Stoma opened at major resection	0 = no, 1 = yes	Number	Derived by the UK Colorectal Cancer Intelligence Hub from information in the relevant hospital discharge dataset
Stoma still open at 18 months following major resection	0 = closed, 1 = open	Number	Derived by the UK Colorectal Cancer Intelligence Hub from information in the cancer registry and relevant hospital discharge dataset
Approach to major resection	0 = open, 1 = laparoscopic	Number	Derived by the UK Colorectal Cancer Intelligence Hub from information in the relevant hospital discharge dataset
Converted laparoscopic procedures	0 = not converted, 1 = converted, 9 = not laparoscopic	Number	Derived by the UK Colorectal Cancer Intelligence Hub from information in the relevant hospital discharge dataset
Type of laparoscopic procedure	0 = standard laparoscopic, 1 = robotic laparoscopic	Number	Derived by the UK Colorectal Cancer Intelligence Hub from information in the relevant hospital discharge dataset
Return to theatre	Indicator of whether an individual had an emergency return to theatre following their major resection within 30 days of surgery	Number	Derived by the UK Colorectal Cancer Intelligence Hub from information in the relevant hospital discharge dataset
Reason for return to theatre	Reason for the return to theatre		Derived by the UK Colorectal Cancer Intelligence Hub from information in the relevant hospital discharge dataset
Failure to rescue	Death within 30 days of the return to theatre	Number	Derived by the UK Colorectal Cancer Intelligence Hub from information in the relevant hospital discharge dataset
Consultant listed as overseeing the episode of care associated with the major resection		Char(9)	
Specialty of overseeing consultant	1 = colorectal 2 = vascular 3 = breast/endocrine	Number	Derived by the UK Colorectal Cancer Intelligence Hub from information in the relevant hospital discharge dataset
	4 = upper gastrointestinal/hepatobiliary/bariatric		
	5 = transplant		
	6 = general surgery 9 = other/unknown		
ACPGBI membership status of consultant	0 = non-member of the ACPGBI 1 = member of the ACPGBI	Char(1)	Derived by the UK Colorectal Cancer Intelligence Hub from information in the relevant hospital discharge dataset
Geography of diagnosis	Option to provide any geography listed at LOCATION based on the postcode of residence of the patient at the time of diagnosis of the colorectal tumour Derived as per user requirements		Derived by the UK Colorectal Cancer Intelligence Hub from information in multiple datasets
Geography of diagnosis and treatment	Option to provide any geography listed at LOCATION based on the hospitals of diagnosis and treatment of the patient at any time in their pathway of care. Derived as per user requirements		Derived by the UK Colorectal Cancer Intelligence Hub from information in multiple datasets
Travel time	Option to provide road travel time for each patient between their home and relevant hospitals they attended at any point across the care pathway. Derived as per user requirements		Derived by the UK Colorectal Cancer Intelligence Hub from information in multiple datasets
Travel distance	Option to provide road travel distance for each patient between their home and relevant hospitals they attended at any point across the care pathway. Derived as per user requirements		Derived by the UK Colorectal Cancer Intelligence Hub from information in multiple datasets
Neoadjuvant rectal cancer treatment	0 = no radiotherapy 1 = short course radiotherapy with immediate surgery	Number	Derived by the UK Colorectal Cancer Intelligence Hub from information in multiple datasets
	2 = short course radiotherapy with delayed surgery		
	3 = long course chemoradiotherapy		
	4 = postoperative radiotherapy		
	5 = other radiotherapy		
Adjuvant chemotherapy	0 = no 1 = yes	Number	Derived by the UK Colorectal Cancer Intelligence Hub from information in multiple datasets
Type of chemotherapy	1 = single agent, 2 = combination agent	Number	Derived by the UK Colorectal Cancer Intelligence Hub from information in multiple datasets
Linkages	Flags to indicate whether the person or tumour links to other datasets available in CORECT-R		Derived by the UK Colorectal Cancer Intelligence Hub from information in multiple datasets

ICD, International Classification of Diseases; NCRAS, National Cancer Registration and Analysis Service; CORECT-R, COloRECTal cancer data Repository; TNM, Cancer staging system (T = Tumour, N = Nodes, M = Metastases); HES, Hospital Episodes Statistics; OPCS4, Classification of Interventions and Procedures version 4; ACPGBI, Association of Coloproctologists of Great Britain and Ireland.

**Table 2:** Variables in the National Anal Cancer Dataset within CORECT-R. Currently these relate to the English population, but work is under way to expand to cover the UK

Data item	Description of field content	Format	Source of information
Pseudonymized person ID	Unique study specific identifier for each patient.	Number	Derived by the UK Colorectal Cancer Intelligence Hub
Pseudonymized tumour ID	Unique study specific identifier for each tumour	Number	Derived by the UK Colorectal Cancer Intelligence Hub
Age	Option to derive age information in a format suitable for the research project needs. Can be single age or summarized in age bands (e.g. 5-year) and can could also include month and year of birth	Number	Cancer registration dataset
Sex	0 = not known, 1 = male, 2 = female, 9 = not specified	Number	Cancer registration dataset
Ethnic group	Option to group ethnicities (e.g. White/non-White/unknown). Several methods of deriving ethnicity information have been used previously and can be derived as per user requirements	Text	Derived by the UK Colorectal Cancer Intelligence Hub from information in the cancer registration and hospital discharge datasets
Socioeconomic status	Option to include an appropriate indicator of socioeconomic status such as the Index of Multiple Deprivation	Number	Derived by the UK Colorectal Cancer Intelligence Hub from information in multiple datasets
Vital status of the patient	A = alive, D = dead, X = exit posting	Char(1)	Cancer registration dataset
Days from diagnosis to death	Time in days between specified anal cancer diagnosis and death or censoring	Number	Derived by the UK Colorectal Cancer Intelligence Hub from information in the cancer registration dataset
Days from another event to date of death	Option to provide number of days from another event to death (e.g. days from diagnosis to death). Derived as per user requirements	Number	Derived by the UK Colorectal Cancer Intelligence Hub from information in key events from different datasets
Cause of death	Option to derive summary cause of death information based on the causes of death listed on death certificates or coded by former regional registries. Derived as per user requirements	Text	Cancer registration dataset
Place of death	Option to derive place of death based on information recorded on death certificates or from Hospital Episode Statistics. Derived as per user requirements		Cancer registration dataset
Diagnosis date	Option to derive diagnosis date in a format suitable for the research project needs. This is likely to be in the format MM/YYYY unless permissions for access to identifiable data have been granted		Cancer registration dataset
Days from another event to date to diagnosis	Option to provide number of days from another event to diagnosis (e.g. days from birth to diagnosis). Derived as per user requirements	Number	Derived by the UK Colorectal Cancer Intelligence Hub from information in multiple datasets
Basis of diagnosis of the tumour	0 = death certificate, 1 = clinical, diagnosis made before death without (2-7), 2 = clinical investigation, 4 = specific tumour markers, 5 = cytology, 6 = histology of metastases, 7 = histology of primary tumour, 9 = unknown	Number	Cancer registration dataset
Route to diagnosis	1 = GP referral, 2 = 2-week wait pathway, 3 = emergency presentation, 4 = other outpatient, 5 = screen detected, 6 = inpatient elective, 7 = death certificate only, 9 = unknown	Number	Cancer registration dataset
Site of neoplasm (4-character code)	Valid 4-digit ICD-10 codes in the range C21	Char(4)	Cancer registration dataset
Morphology	The original 5-digit ICD03 morphology information captured by NCRAS	Char(5)	Cancer registration dataset
Morphology group	A grouped morphology variable derived by grouping the detailed morphology information available in NCRAS to provide the more useable histology types. For many analyses it may be appropriate to limit cases to exclude rare morphological sub types with non-standard treatment or unusual behaviour.	Number	Derived by the UK Colorectal Cancer Intelligence Hub from information in the cancer registration dataset
Summary stage	A summary stage that combines information from the multiple staging variables available in NCRAS to maximize the number of cases with a stage classification	Number	Cancer registration dataset
Staging information	Option to derive staging information from any of the data items available in the CORECT-R tables (likely to be predominantly NCRAS Tumour table). This can include individual components of TNM or any other information that can be reported alone or combined as demanded by the research project. Derived as per user requirements		Derived by the UK Colorectal Cancer Intelligence Hub from information in the cancer registration dataset
Comorbidity scores	Option to access a number of different comorbidity scores (such as Charlson, Elixhauser, C3) created as per user requirements. This could include using a previously published application of these scores or bespoke algorithms as demanded by the research project	Number	Derived by the UK Colorectal Cancer Intelligence Hub from information in multiple datasets
Abdominoperineal excision (APE)	1 = APE, 0 = no APE	Char(1)	Derived by the UK Colorectal Cancer Intelligence Hub from information in the cancer registry and relevant hospital discharge dataset
Days from diagnosis to APE	Time in days between specified anal cancer diagnosis and APE	Number	Derived by the UK Colorectal Cancer Intelligence Hub from information in the cancer registry and relevant hospital discharge dataset
Surgical information	Option to provide surgical treatment information. Derived as per user requirements		Derived by the UK Colorectal Cancer Intelligence Hub from information in multiple datasets
Radiotherapy information	Option to provide radiotherapy treatment information. Derived as per user requirements		Derived by the UK Colorectal Cancer Intelligence Hub from information in multiple datasets
Chemotherapy information	Option to provide chemotherapy treatment information. Derived as per user requirements		Derived by the UK Colorectal Cancer Intelligence Hub from information in multiple datasets
Linkages	Flags to indicate whether the person or tumour links to other datasets available in CORECT-R		Derived by the UK Colorectal Cancer Intelligence Hub from information in multiple datasets

ICD, International Classification of Diseases; NCRAS, National Cancer Registration and Analysis Service; CORECT-R, COloRECTal cancer data Repository; TNM, cancer staging system (T = Tumour, N = Nodes, M = Metastases); APE, abdominoperineal excision.

The CORECT-R system also provides a secure Trusted Research Environment (TRE) via which researchers can access data. The CORECT-R TRE is a secure analytical area that, following both project and user approval, is accessed via two-factor authentication and a virtual desktop. It contains database and statistical analysis software and users can also bring their own software in to the environment once approved. The TRE infrastructure is provided by the company AIMES and hosted in partnership with Cancer Research UK.

## Data resource use

The potential of the data in CORECT-R is huge, enabling research into all aspects of the disease from its aetiology to its management and outcome. Ethical approval to establish a Research Database was obtained (UK NHS Health Research Authority 18/SW/0134) and this supports the use of its contents for projects that will promote early diagnosis, help quantify and address inequalities, support cancer research and improve outcomes. The Scottish Public Benefit Privacy Panel has also approved an initial phase of the Programme (PBPP: 1718–0026), with data accessible to the current project team. If a researcher has a project beyond these approved uses, the Hub will actively support applications to extend the use of the resource.

An example of how the resource has been used is the investigation of post-colonoscopy colorectal cancers (PCCRCs). These can occur when the main diagnostic test used to identify the disease, colonoscopy, fails to detect the tumour or the precursor lesion. Via linkage and analysis of cancer registry, hospital and screening data (combined in CORECT-R), work has been undertaken to identify PCCRCs and understand their occurrence across the English NHS.^7^^,^^26^ These studies have helped to identify groups of individuals at greater risk of developing a PCCRC and also colonoscopy providers with significantly higher, and lower, PCCRC rates.^7^ To try and ensure this intelligence is used to inform efforts to reduce rates, the results of the study were disseminated in the peer-reviewed literature and to all colonoscopy providers via the Getting it Right First Time (GIRFT) programme.^27^ In addition, all providers with outlying rates, in both a positive and negative direction, were directly informed of their results. This led many to seek to review their cases to understand why they arose. The CORECT-R team supported and facilitated their applications to the Office for Data Release at Public Health England to identify their PCCRC cases and to audit their colonoscopy services.

The resource is routinely used to quantify variations in colorectal cancer care across the NHS. For example, the Yorkshire Cancer Research Bowel Cancer Improvement Programme^28^ has used the data to produce annual reports quantifying variation in the patient (sex, age, socioeconomic status and comorbidity) and tumour (stage at diagnosis, site of tumour and mode of presentation) characteristics of the colorectal cancer populations treated by each of Yorkshire’s multidisciplinary teams, as well as variation in management and outcome. Treatments examined include use and type of major resection as well as the approach to surgery, use of neoadjuvant radiotherapy^19^ and adjuvant chemotherapy.^21^ Significant work has also been undertaken looking at the management of metastatic disease.^20^ The Programme team regularly provide these reports to Yorkshire’s multidisciplinary teams giving them information on how their cases compare with those managed by other teams in the region, as well as England as a whole. Clinical engagement is then sought to try to understand and explain why any variation exists which, in turn, leads to initiatives aimed at minimizing it.

There are numerous other examples of how the data within CORECT-R can be used and more details are available on the Hub’s website, alongside a summary of all the projects currently under way, or completed, using the resource.

## Strengths and weaknesses

The main strength of CORECT-R is that it streamlines the extremely resource-intensive processes that researchers go through to access population-based datasets relevant to colorectal cancer. Previously, many research and service groups all worked in parallel to seek their own permissions to obtain extracts of data. They then undertook bespoke linkages and used different methods to analyse these data and, in consequence, often obtained slightly different findings.^29^ This results in significant duplication of effort and resource, as well as confusion for those wishing to make use of the resulting intelligence. In addition, multiple data transfers increase the risk of data breaches that may threaten patient confidentiality and public trust. CORECT-R offers an alternative and more efficient route to the data and this collaborative approach, in turn, enhances the abilities of the cancer intelligence community to produce the evidence needed to drive improvements in colorectal cancer care.

Although the collaborative approach of CORECT-R is a strong model, it does pose challenges. These often arise because the resource aims to align datasets owned by multiple different organizations who all have their own policies and procedures relating to data access and these sometimes conflict. In addition, when different datasets containing the same information for individuals (for example date of diagnosis, type of surgery or site of tumour) are aligned, they can disagree, and this leads to significant challenges in quality-assuring the information. Again however, via the collaborative nature of the Hub and the transparent data management and processing pathways it adopts, these challenges can be overcome.

CORECT-R aims to contain detailed information about all colorectal cancer patients in the UK and it is vital to respect the interests of those people to whom these data pertain and, in a very real sense, belong. Another strength of the UK Colorectal Cancer Intelligence Hub and its CORECT-R resource is its involvement of patients and the public. As a direct result, the concerns about the security and use of patient data are fully appreciated by the Hub team, and we have sought to design a system that will minimize these risks and anxieties while also maximizing the benefit the analysis of such population data can have. The Hub’s Patient-Public Group is extremely active and involved in the management of CORECT-R and its outputs.

## Data resource access

Researchers who wish to make use of the CORECT-R resource should contact the UK Colorectal Cancer Intelligence Hub team and discuss their requirements. The application process will differ depending on the details of the proposed project and the source of the data required. The application process centres on the development of a project protocol that describes the information required, justifies its use and sets out the study objectives. The Hub team will support the applicant through the process. For more details, see [https://www.ndph.ox.ac.uk/corectr].

The Hub website also hosts information on the data available within CORECT-R. The data catalogue contains detailed information on the ‘research ready’ national colorectal and anal cancer datasets. In addition, it also provides information on all the individual component datasets within CORECT-R and where to find more information about their content.

Cancer Research UK, the funders of the resource, and the UK Colorectal Cancer Intelligence Hub itself, are keen to ensure that the data within CORECT-R are used for the maximum benefit of colorectal cancer patients and, as such, have resourced the project to enable free access to academic researchers.

The publications arising from any projects that include CORECT-R data have to acknowledge the resource and its funding as well as the people who contribute data into the resource, with the following attributions:

This project involves data that have been provided by, or derived from, patients and collected by the NHS as part of their care and support.

This work was supported by Cancer Research UK (C23434/A23706), which underpinned data access via the UK Colorectal Cancer Intelligence Hub.

Finally, CORECT-R contains many different datasets and, if used in a particular project, the organizations who have contributed data need to be acknowledged. Users will be made aware of relevant attributions on a project-by-project basis.

## Funding

The CORECT-R resource is supported by Cancer Research UK (grant C23434/A23706).

## Conflict of Interest

None declared. 
